# Timing of in utero malaria exposure influences fetal CD4 T cell regulatory versus effector differentiation

**DOI:** 10.1186/s12936-016-1545-6

**Published:** 2016-10-07

**Authors:** Mary Prahl, Prasanna Jagannathan, Tara I. McIntyre, Ann Auma, Lila Farrington, Samuel Wamala, Mayimuna Nalubega, Kenneth Musinguzi, Kate Naluwu, Esther Sikyoma, Rachel Budker, Hilary Vance, Pamela Odorizzi, Patience Nayebare, John Ategeka, Abel Kakuru, Diane V. Havlir, Moses R. Kamya, Grant Dorsey, Margaret E. Feeney

**Affiliations:** 1 Department of Pediatrics, University of California-San Francisco, 3333 California Street, Suite 315, Box 1234, San Francisco, CA 94143 USA; 2 Department of Medicine, University of California-San Francisco, 3333 California Street, Suite 315, San Francisco, CA 94143 USA; 3 Infectious Diseases Research Collaboration, 2C Nakasero Hill Road, PO Box 7475, Kampala, Uganda; 4 School of Medicine, College of Health Sciences, Makerere University of College of Health Sciences, PO Box 7072, Kampala, Uganda

**Keywords:** Pregnancy-associated malaria, Fetal immune response, Immune tolerance, CD4 T cells, Dendritic cells, Loop-mediated isothermal amplification

## Abstract

**Background:**

In malaria-endemic areas, the first exposure to malaria antigens often occurs in utero when the fetal immune system is poised towards the development of tolerance. Children exposed to placental malaria have an increased risk of clinical malaria in the first few years of life compared to unexposed children. Recent work has suggested the potential of pregnancy-associated malaria to induce immune tolerance in children living in malaria-endemic areas. A study was completed to evaluate the effect of malaria exposure during pregnancy on fetal immune tolerance and effector responses.

**Methods:**

Using cord blood samples from a cohort of mother-infant pairs followed from early in pregnancy until delivery, flow cytometry analysis was completed to assess the relationship between pregnancy-associated malaria and fetal cord blood CD4 and dendritic cell phenotypes.

**Results:**

Cord blood FoxP3^+^ T_reg_ counts were higher in infants born to mothers with *Plasmodium* parasitaemia early in pregnancy (12–20 weeks of gestation; p = 0.048), but there was no association between T_reg_ counts and the presence of parasites in the placenta at the time of delivery (by loop-mediated isothermal amplification (LAMP); p = 0.810). In contrast, higher frequencies of activated CD4 T cells (CD25^+^FoxP3^−^CD127^+^) were observed in the cord blood of neonates with active placental *Plasmodium* infection at the time of delivery (p = 0.035). This population exhibited evidence of effector memory differentiation, suggesting priming of effector T cells in utero. Lastly, myeloid dendritic cells were higher in the cord blood of infants with histopathologic evidence of placental malaria (p < 0.0001).

**Conclusion:**

Together, these data indicate that in utero exposure to malaria drives expansion of both regulatory and effector T cells in the fetus, and that the timing of this exposure has a pivotal role in determining the polarization of the fetal immune response.

## Background

More than 12.4 million pregnancies occur annually in regions at risk of malaria transmission [1], and one in four pregnant women in sub-Saharan Africa have evidence of infection with malaria at parturition [2]. Pregnancy-associated malaria results in tremendous obstetrical and paediatric morbidity, including maternal anaemia, intra-uterine growth retardation, low birth weight, prematurity, miscarriage, and stillbirth. It has been estimated that pregnancy-associated malaria causes 100,000 infant deaths per year [2]. In addition to these immediate consequences, exposure to malaria antigens in utero may influence the developing fetal immune system in ways that remain poorly defined.

Several studies have reported that infants born to mothers with placental malaria (PM) are themselves at higher risk for malaria during the first few years of life [3–5]. While this may be due in part to the fact that mothers and their infants experience similar levels of exposure to infected mosquitoes, recent studies suggest that tolerance to malaria antigens may be induced following exposure in utero [6–13], suggesting a potential immunologic explanation for this association. Infant T cells are uniquely predisposed toward the induction of tolerance upon encounter with foreign antigens, presumably as a result of the requirement to maintain maternal-fetal tolerance [14]. In malaria-endemic areas, many infants are first exposed to malaria antigens in utero, during a critical period of fetal immune development. In utero exposure to malaria has been reported to induce numerous immunoregulatory mechanisms in the fetus, including expansion of FoxP3^+^ T regulatory cells, increased levels of suppressive cytokines such as IL-10 and TGF-β and diminished CD4 Th1 responses [6, 7, 10, 12, 13]. Moreover, one study found that some exposed infants acquire a tolerant phenotype that is associated with an increased risk of malaria infection later in childhood [6]. While it is widely accepted that CD4 T cells in the neonate respond differently to antigen compared to adults [15], including skewing toward Th2 and T_reg_ differentiation [16], the biologic mechanisms underlying these differences are unclear. These differences may arise from T cell-intrinsic factors, such as epigenetic programming [17] or differences in fetal haematopoietic stem-progenitor cells [16], or as a result of insufficient priming by immature dendritic cells.

Prior studies comparing cord blood CD4 T regulatory cell (T_reg_) frequencies in infants with and without in utero malaria exposure have yielded conflicting results. Some studies have observed higher frequencies of T_regs_ in placenta malaria-exposed infants [9–11], while others have not [7, 8, 12]. This may be due to several factors. First, past studies differ in their gating strategies to quantify T_regs_, with earlier studies including all CD25^+^ CD4 T cells, while later studies used more stringent definitions including expression of the transcription factor FoxP3 and/or low expression of CD127. PM diagnosis and categorization also differed in prior studies. Most were cross-sectional and evaluated for malaria infection only at the time of delivery, a strategy that may have missed maternal infections that occurred earlier in pregnancy. Finally, PM was diagnosed in some studies based on placental blood smear while others used more sensitive histopathologic criteria. To date, no studies have assessed the relationship of cord blood regulatory CD4 T cell frequencies to placental infection using sensitive nucleic acid tests for *Plasmodium falciparum*.

This study evaluated the impact of in utero malaria exposure on the frequency and phenotype of CD4 T regulatory cells and dendritic cells, using cord blood samples from infants born in a highly malaria-endemic region of Uganda. Mother-infant pairs were followed longitudinally beginning from 12 to 20 weeks gestation through delivery. CD4 T cells were compared in infants born to mothers with and without evidence of *Plasmodium* infection during pregnancy to test the hypothesis that in utero malaria exposure would result in an expansion of fetal regulatory CD4 T cells and/or effector CD4 T cells.

## Methods

### Ethical approval

Informed consent was obtained from the parent or guardian of all study participants. The study protocol was approved by the Uganda National Council of Science and Technology (UNCST), and the institutional review boards of the University of California, San Francisco (UCSF) and Makerere University.

### Study site and participants

Samples were collected from a clinical trial of prenatal malaria chemoprevention conducted in Tororo, Uganda, an area of high malaria endemicity. Clinical trial outcomes are described in a prior publication [18]. Briefly, 300 HIV-negative mother-infant pairs were enrolled between 12 and 20 weeks of gestation (June to October 2014). Study clinicians performed ultrasound dating on all participants to determine gestational age at the time of enrolment. Evaluated enrollees were randomized to standard malaria chemoprevention (three-dose sulfadoxine-pyrimethamine) versus enhanced malaria chemoprevention (monthly dihydroartemisinin-piperaquine) from which sufficient cord blood mononuclear cells (CBMCs) were available. Participants randomized to the standard chemoprevention arm were administered sulfadoxine-pyrimethamine at 20, 28 and 36 weeks of gestation. Participants randomized to the monthly dihydroartemisinin-piperaquine arm received drug every 4 weeks beginning at 16 or 20 weeks based on gestational age at enrolment. At enrolment, study participants received an insecticide-treated bed net. All mothers received one dose of mebendazole in the second trimester per Ugandan Ministry of Health guidelines. Participants received their routine medical care at the study clinic and had routine laboratory assessments completed every 4 weeks. Enrollees were encouraged to deliver at the study site hospital.

### Clinical outcomes

Mothers were evaluated throughout pregnancy for *Plasmodium* parasitaemia beginning at enrolment (12–20 weeks of gestational age), and additionally with routine monthly surveillance testing peripheral blood via loop-mediated isothermal amplification (LAMP) kits (Eiken Chemical) which detect *Plasmodium* DNA [18, 19]. During febrile episodes mothers were evaluated with blood microscopy, and if positive, treated per local guidelines for clinical malaria, as previously described [18].

At the time of delivery, maternal peripheral blood, placental blood and cord blood was tested for parasitaemia by both LAMP and microscopy. Placental tissue was processed for histopathologic evidence of malaria infection, determined by standardized placental malaria histopathology criteria as previously described [18, 20, 21].

### CBMC collection

At the time of delivery, whole cord blood was collected in umbilical cord blood collection kits (Pall Medical). Whole blood was additionally collected in EDTA tubes for fresh whole cord blood experiments. CBMCs were isolated by Ficoll-histopaque density centrifugation (GE Life Sciences). CBMCs were cryopreserved in liquid nitrogen and transported for analysis in San Francisco, CA, USA. Post-thaw CBMC viability was analysed via Millipore cell counter and was consistently >78 %.

### Flow cytometry immunophenotyping

CBMCs were thawed, aliquoted at 1 × 10^6^ cells, surface and intracellularly stained using standard protocols using the following antibodies: allophycocyanin/Cy7 (APC/Cy7)-conjugated CD3 (clone OKT3), peridinin chlorophyll protein (PerCP)-conjugated CD4 (clone RPA-T4), Brilliant Violet 421-conjugated CD25, Brilliant Violet 650-conjugated CD127, Brilliant Violet 605-conjugated CD45RO, allophycocyanin (APC)-conjugated CCR4, fluorescein isothiocyanate (FITC)-conjugated CCR7 (BioLegend), phycoerythrin-Cy7 (PE-Cy7)-conjugated CD95 (BD Pharmingen), and phycoerythrin (PE)-conjugated FoxP3 (eBioscience). Brilliant Violet 510-conjugated CD8, Brilliant Violet 510-conjugated CD14, Brilliant Violet 510-conjugated CD19 (BioLegend) and LIVE/DEAD aqua amine (Invitrogen) were included to exclude non-specific binding and to isolate the CD4 cell population. To normalize the frequency of analysed CD4 sub-sets (T_regs_ and CD127^+^ cells) from cryopreserved CBMCs to absolute rates of CD4 per microlitre of fresh whole cord blood, absolute CD4 sub-set counts were calculated (CD4 sub-set frequency * absolute CD4 count per microlitre of whole cord blood).

**Fig. 1:**
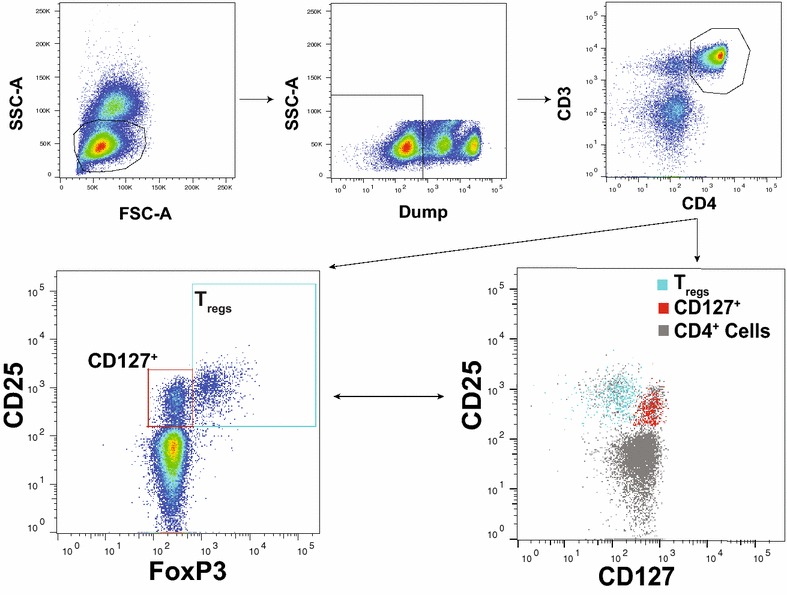
Flow cytometry gating strategy. Flow cytometric analysis of cord blood live CD4^+^CD25^+^ T cells revealed two distinct sub-populations based on FoxP3 and CD127 expression, T regulatory (CD4^+^CD25^+^FoxP3^−^CD127^lo/−^) and CD127^+^ (CD4^+^CD25^+^FoxP3^−^CD127^hi^) cells

Flow cytometry data were collected on an LSR2 four-laser flow cytometer (Becton Dikinson) with FACSDiva software. Colour compensations were performed using compensation beads. Fluorescence-minus-one samples were used to define negative and positive populations for CD95, CD45RO, CCR4, and CCR7. An isotype control was used to define negative and positive populations for FoxP3 and CD25. Cellular profiles were gated on live CD3^+^CD4^+^ lymphocytes (Fig. 1).

### Absolute whole blood immune cell counts

Dendritic cells and CD4 T cells were enumerated from 50 μL of whole cord blood stained with antibodies in BD TruCount™ tubes or with 20 μL of CountBright™ counting beads (ThermoFisher Scientific). Cells were incubated 20 min, and 900 μL of BD FACS lysis solution was added for 15 min. CD4 T cell staining was performed on 152 cord blood samples using PerCP-conjugated CD3, APC-conjugated CD4 antibodies. Dendritic cell staining was performed on 145 cord blood samples using FITC-conjugated Lin-2 (*CD3, CD14, CD19, CD20, CD56*), PE-conjugated CD123, PerCP-conjugated HLA-DR, and APC-conjugated CD11c (BD Pharmingen). Cells were immediately analysed on an Accuri A6 cytometer. Dendritic cells were defined as Lin-2^−^HLA-DR^+^, myeloid dendritic cells were defined as Lin-2^−^HLA-DR^+^CD11c^+^CD123^−^, plasmacytoid cells were defined as Lin-2^−^HLA-DR^+^CD11c^−^CD123^+^.

### Statistical methods

Statistical analyses were performed using PRISM 6.0 (GraphPad) and STATA 13.1 (StataCorp). Non-parametrically distributed cellular frequencies were log-transformed for normalization. Associations between in utero malaria exposure and cellular frequencies were compared using the Wilcoxon rank sum and/or Student’s t test as appropriate. Associations between continuous variables were compared using Spearman’s rank correlation (r_s_). Two-sided p values were calculated for all test statistics, and p < 0.05 was considered significant.

## Results

### Study cohort and pregnancy-associated malaria outcomes

The study cohort consisted of 166 infants born to mothers enrolled in a clinical trial of artemisinin-based prenatal malaria chemoprevention. Women were enrolled at 12–20 weeks of gestation and evaluated monthly for parasitaemia by LAMP until delivery. Overall 79 % (131/166) of enrollees had some evidence of pregnancy-associated malaria (any *Plasmodium* detected during pregnancy or delivery). At the time of enrolment, 54 % of mothers were parasitaemic by LAMP, suggesting a high burden of infection early in pregnancy. Based on routine monthly surveillance, 49 % of mothers were parasitaemic by LAMP at least once after enrolment and prior to delivery. At delivery, placental specimens were assessed for evidence of PM by histopathology (using standardized criteria by Rogerson et al. [21]), placental blood microscopy, and placental blood LAMP. Histopathologic evidence of infection was observed in 38 % (63/166) of placentas, but only 12 % (20/166) of placental blood samples were positive by LAMP (Table 1). The majority of placentas with histopathologic evidence of malaria infection had only pigment deposition without parasites, inflammatory infiltrate, or positive LAMP, suggesting a remote malaria infection. Among women randomized to artemisinin-based prenatal chemoprevention, the rate of at least one episode of parasitaemia during the trial period decreased by fourfold (p < 0.001), the rate of histopathologic evidence of infection was decreased by twofold (p = 0.001), and the rate of placental blood LAMP infection decreased by sixfold (p < 0.001) [18].

**Table 1:** Cohort characteristics and malaria outcomes

Maternal characteristics	n (%)
Total enrollees	166
Maternal age (mean)	22.1
Previous pregnancies (%)	
0	56 (33.7)
1	51 (30.7)
2 or more	59 (35.5)
Randomized chemoprevention arm	
Dihydroartemisinin-piperaquine	86 (51.8)
Sulfadoxine-pyrimethamine	80 (48.2)

Malaria outcomes	Positive n (%)
Assessments during pregnancy	
LAMP^u^ at enrolment	89 (53.6)
Any LAMP during monthly screening	81 (48.8)
Assessments at delivery	
Maternal peripheral blood	
Microscopy	4 (2.4)
LAMP	20 (12.1)
Placental blood	
Microscopy	4 (2.4)
LAMP	20 (12.1)
Cord blood	
Microscopy	0 (0)
LAMP	3 (1.8)
Histopathology	63 (38.0)
Parasites, pigment in monocytes ± fibrin	4 (2.4)
Pigment only	59 (35.5)
Any malaria during pregnancy or delivery	131 (78.9)

Infant outcomes	n (%)
Preterm (<37 weeks)	9 (5.4)
Low birth weight (<2500 g)	9 (5.4)

^u^Loop-mediated isothermal amplification

### Cord blood FoxP3^+^ T_reg_ counts are higher in infants born to mothers with parasitaemia early in pregnancy

**Fig. 2:**
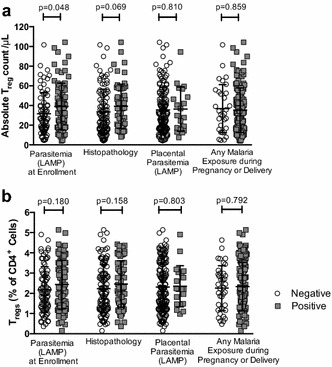
Infants born to mothers with parasitaemia early in gestation have higher cord blood T_reg_ counts. **a** Absolute T_reg_ counts were higher in infants born to mothers with parasitaemia at time of enrolment (12–20 weeks gestation) by Wilcoxon ranksum testing (n = 152); **b** frequency of cord blood T_regs_ were not significantly associated with malaria exposure outcomes by Wilcoxon rank sum testing (n = 166)

Elevated frequencies of cord blood T regulatory cells have been reported among infants born to mothers with PM in some, but not all, prior studies [7–12]. A stringent definition of T regulatory cells was used, defined as CD25^+^FoxP3^+^CD127^lo/−^ CD4 T cells (Fig. 1), and compared in utero malaria exposed and unexposed infants. Both T_reg_ frequency (as a percentage of all CD4 T cells) and the absolute counts of T_regs_ per microlitre of fresh whole cord blood were assessed. Infants born to mothers with parasitaemia by LAMP at the time of enrolment (12–20 weeks gestation) had significantly higher absolute counts of T_regs_ compared with infants whose mothers were not parasitaemic (38.9 vs 31.7 cells/μL whole cord blood p = 0.048; Fig. 2). Frequencies of T_regs_ were also higher in this group, although this difference was not statistically significant (p = 0.180). However, there was no association between the frequency of T_regs_ and the presence of parasites in the placenta at the time of delivery (by LAMP; p = 0.803). Placental histopathology reflects both long-standing chronic malaria infection, represented by pigment deposition, as well as active malaria infection, represented by parasites and inflammation [21]. Both the frequencies and absolute counts of T_regs_ were higher in infants whose mothers had histopathologic evidence of infection, but this difference was not statistically significant (p = 0.158 and p = 0.069, respectively; Fig. 2). Lastly, the frequency of T_regs_ was not associated with the composite outcome of ‘any malaria exposure’ during pregnancy or delivery (p = 0.792). Cord blood T_reg_ frequency was not associated with gravidity (p = 0.281), prematurity (p = 0.474), low birth weight (p = 0.973), nor with randomized chemoprevention arm (p = 0.283). These data suggest that in utero malaria antigen exposure early in pregnancy, but not active placental infection late in pregnancy, is associated with an expansion of fetal T_regs._

### Frequencies of activated CD127^+^ CD4 T cells are increased in infants with active placental infection

**Fig. 3:**
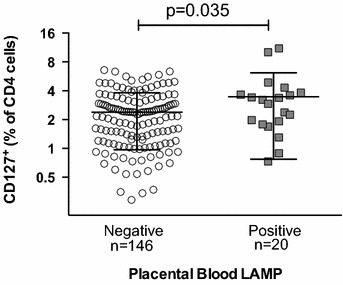
Cord blood CD25^+^FoxP3^−^CD127^+^ CD4 T cell frequency is higher in placental *Plasmodium* LAMP infection. Frequencies of cord blood CD4^+^CD25^+^FoxP3^−^CD127^+^ cells are higher in infants with positive placental blood LAMP test indicating presence of *Plasmodium* DNA in placental blood (Student’s t test; n = 166)

Several prior studies reporting an association between regulatory T cells and PM had defined T_regs_ as all CD4 T cells with high expression of CD25. However, it is now known that CD25 is expressed on a variety of activated CD4^+^ cells that lack regulatory function and do not express the canonical T_reg_ transcription factor FoxP3. Using additional T_reg_ markers (CD127 and FoxP3), this study found that CD25^hi^ CD4 T cells can be sub-divided into two major populations: conventional T_regs_ (CD25^+^FoxP3^+^CD127^lo/−^) and putatively activated CD127^+^ CD4 T cells (CD25^+^Foxp3^−^CD127^+^; Fig. 1). This latter population phenotypically resembles effector memory T cells (T_EM_) which have recently been described in healthy fetuses even in the absence of any infectious exposures [22]. Notably, infants with active placental LAMP infections had significantly higher frequencies of these activated CD127^+^ cells (p = 0.035; Fig. 3). However, the frequency of activated CD127^+^ cells was not elevated in infants born to mothers with histopathologic evidence of PM (p = 0.656), parasitaemia at enrolment (p = 0.752), or in infants born to mothers with parasitaemia between 20 and 40 weeks of gestation (p = 0.315). Furthermore, the frequency of activated CD127^+^ cells in cord blood was not associated with gravidity (p = 0.951), prematurity (p = 0.455), low birth weight (p = 0.405), nor with randomized chemoprevention arm (p = 0.339). Together, these data suggest that in utero malaria exposure early in pregnancy may drive a regulatory response, while late exposure to parasites present at the time of delivery may drive an activated effector CD4 T cell response.

### Cord blood activated CD127^+^ CD4 T cells exhibit evidence of effector memory differentiation

**Fig. 4:**
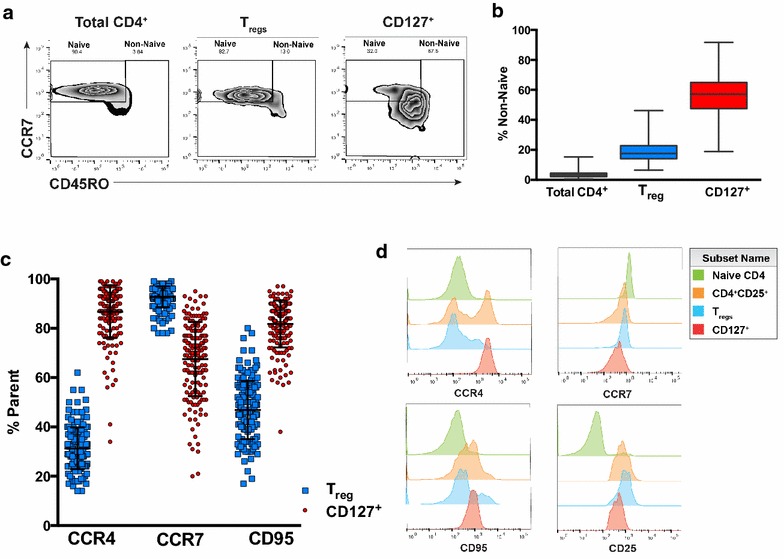
Memory and activation marker expression by CD127^+^ CD4 cells. **a** Expression of CCR7 and CD45RO expression on total CD4 cells, T_regs_ and CD127^+^ CD4 cells; **b** percentage of CD4^+^ T cells, T_regs_, and CD127^+^ CD4 cells that are phenotypically non-naïve by CCR7/CD45RO gating (n = 166); **c** percentage of T_regs_ vs CD127^+^ cells expressing cell surface markers (n = 166); **d** distinct expression of cell surface markers CCR4, CCR7, and CD95 by all CD4^+^CD25^+^ T cells, T_regs_, and CD127^+^ cells in one representative cord blood sample

Given their divergent associations with early and late gestation infections, additional cell surface markers were assessed to further distinguish T_regs_ (CD25^+^FoxP3^+^CD127^lo/−^) and activated CD127^+^ (CD25^+^FoxP3^−^CD127^hi^) sub-sets of cord blood CD25^+^ CD4 T cells. While the majority of cord blood CD4 T cells, as well as the T_reg_ (CD25^+^FoxP3^+^CD127^lo/−^) sub-set, were CD45RO^−^CCR7^+^, indicating a naïve phenotype, most activated CD127^+^ cells had up-regulated CD45RO and down-regulated CCR7 expression (Fig. 4), suggesting effector-memory differentiation. In addition to down-regulation of the lymph node migration marker CCR7, activated CD127^+^ CD4 T cells exhibited striking up-regulation of CCR4, a cell surface marker that has been associated with Th2 differentiation, as well as up-regulation of CD95, a cell surface protein that mediates apoptosis (Fig. 4c). Thus, T_regs_ (CD25^+^FoxP3^+^CD127^lo/−^) and activated CD127^+^ (CD25^+^FoxP3^−^CD127^hi^) CD4 T cells exhibited markedly distinct expression of several cell surface molecules (Fig. 4d). Based on their memory phenotype differentiation, up-regulation of CD95, and expansion in infants with active placental malaria infection, these data suggest that CD127^+^ CD4 T cells may be an antigen-experienced effector memory cell population.

### Myeloid dendritic cell counts are higher in cord blood of infants with histopathologic evidence of PM

**Fig. 5:**
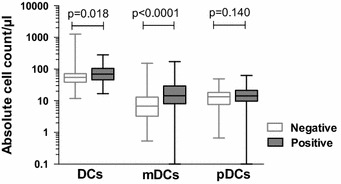
Myeloid dendritic cell counts are higher in placental histopathologic malaria infection. Absolute counts of total dendritic cells (Lin^−^HLA-DR^+^) (DCs), myeloid dendritic cells (Lin^−^HLA-DR^+^CD11c^+^CD123^−^) (mDCs), and plasmacytoid dendritic cells (Lin-HLA-DR^+^CD11c^−^CD123^+^) (pDCs) in cord blood of infants with negative and positive histopathology for placental malaria (Wilcoxon rank sum; n = 145)

The role of antigen-presenting cells was additionally investigated as a potential contributor to the induction of regulatory immune responses. Given the role of dendritic cells in T cell priming [23] and tolerance [24], The absolute count of myeloid and plasmacytoid dendritic cell (mDC and pDC) sub-sets in whole cord blood was measured from 145 infants. Total dendritic cell counts were higher among infants born to mothers with histopathologic evidence of PM (p = 0.018). This difference was driven by higher mDC counts among histopathologic PM-exposed infants (p < 0.0001), as pDC counts were not significantly different (p = 0.139; Fig. 5). Active placental infection (LAMP positive) was not associated with changes in dendritic cell counts, indicating that dendritic cell expansion is more pronounced following remote infection rather than late-gestation infection.

Additionally, the association of dendritic cell sub-sets with regulatory and effector CD4 T cell subsets. pDC counts were positively associated with both absolute T_reg_ counts (p = 0.0139, r_s_ 0.204) and absolute CD127^+^ cell counts (p = 0.027, r_s_ 0.183). mDC counts were not significantly associated with either CD4 sub-set.

## Discussion

Investigations from the presented longitudinal study detail the relationship of cord blood regulatory and activated CD4 T cells to in utero malaria exposure. Findings include that while T_regs_ were higher among infants with early/remote malaria exposure, active placental infection with persistence of plasmodial DNA at the time of delivery was associated with expansion of activated CD127^+^ CD4 T cells resembling effector memory T cells. mDC, critical for T cell priming, were also expanded in PM-exposed infants. These findings suggest that in utero exposure to malaria impacts both fetal T cells and antigen presenting cells, and the timing of malaria infections during pregnancy may have a pivotal role in determining the polarization of the infant immune response.

The role of FoxP3^+^ regulatory CD4 T cells in PM infection has been controversial. Some studies have found elevated cord blood T_reg_ frequencies in the setting of PM [9–11], while others have found no association [7, 8, 12]. Depletion of CD4^+^CD25^+^ T cells (putative T_regs_) has been shown to abrogate malaria specific T-cell cytokine production, suggesting that T_regs_ play a functionally suppressive role in malaria-exposed infants, regardless of whether they are increased in frequency [10].

The longitudinal design of this study provides insight on the effects of timing and type of malaria exposure on fetal T_reg_ induction. Cord blood T_regs_ were associated with maternal parasitaemia early in pregnancy (12–20 weeks gestation), but not with composite malaria exposure (any malaria exposure during gestation) or acute placental infection. Among prior studies assessing the association of T_regs_ with in utero malaria exposure, many evaluated malaria exposure only at the time of delivery. However, women in highly endemic regions can develop PM early in pregnancy and often experience repeated episodes of malaria parasitaemia during pregnancy and at delivery. Moreover, fetal immune development is a dynamic process. High levels of T_reg_ cells are present in the fetal thymus and fetal lymph nodes at 12–20 weeks of gestation [25] and mid-gestation T cells are particularly predisposed to differentiate into tolerogenic T_regs_ following stimulation by antigen [16]. Exposure to malaria antigens during this critical window of immune development could explain the expansion of T regulatory cells following early gestational exposure to malaria antigens.

The definition of T regulatory cells differed greatly among earlier studies, and these differences may have contributed to conflicting findings. For instance, several studies defined T_regs_ as all CD4^+^CD25^hi^ cells, but it has since been appreciated that FoxP3 expression correlates with suppressive function. Prior studies that used less precise definitions (e.g., without assessing expression of transcription factor FoxP3) found associations with in utero malaria exposure [9–11]. In contrast, most studies with more stringent definitions of T_regs_ (including assessment for FoxP3 expression) did not find association between frequency of cord blood T_regs_ and in utero malaria exposure [7, 8, 12]. This study found that CD25^hi^ CD4 T cells can be sub-divided into two distinct populations, FoxP3^+^ T_regs_, which have low CD127 expression, and CD127^+^ cells which lack FoxP3 expression. Prior studies that associated PM with increased frequencies of CD4^+^CD25^+^ cord blood cells would have been unable to differentiate FoxP3^+^ T_regs_ from the activated cord blood CD127^+^ CD4 T cell population that were found to be expanded among infants with active placental infection.

An activated CD127^+^ CD4 T cell cord blood population was observed that phenotypically resembles the T effector memory (T_EM_) cells recently described by Zhang et al. [22]. These T_EM_ cells were shown to have a polyclonal T-cell receptor repertoire, exhibit proliferative capacity, and be poised for inflammatory cytokine production, although they developed in a presumably sterile intra-uterine environment [22]. The expansion of activated T_EM_-like CD127^+^ cells in neonates with active placental infection raises the possibility that these cells may have specificity for malaria antigens, which are known to cross into the fetal circulation during pregnancy-associated malaria [26, 27]. However, further studies are needed to determine whether these are malaria-specific. Prior studies evaluating fetal CD127^+^ CD4 T cells have not found an association with malaria exposure, but in these studies PM was diagnosed based only on less sensitive blood smears [8, 11]. In this study, activated CD127^+^ CD4 cells were found to highly up-regulate CCR4, which has been classically associated with Th2 differentiation. However, cord blood CD4^+^ effector cells are more ambiguous in their Th differentiation and may defy classic Th sub-set differentiation described in adults [22].

Additional findings include that PM exposure was strongly associated with an expansion of cord blood mDCs, which play a central role in priming T cell responses. *Plasmodium falciparum* is known to modulate DC function and maturation, leading to decreased expression of maturation, cell adhesion and co-stimulatory markers. The resulting immature, or ‘tolerized’ DCs have a diminished capacity to activate effector T cells [28]. These results concur with a prior study of Gabonese infants, which found elevated cord blood mDCs but not pDCs in malaria-exposed infants [29]. However, a more recent study assessing cord blood mDCs and pDCs in malaria-exposed infants found no association with PM [30]. In vitro studies of cord blood have shown that dendritic cells can induce T_reg_ differentiation [31] and pDCs induce T_regs_ from CD4 T cells [32, 33] with greater induction capacity than mDCs [24, 34]. In the present study, although pDC counts were not associated with malaria outcomes, they were positively associated with absolute T_reg_ and absolute CD127^+^ cord blood counts. Further mechanistic evaluations are needed to delineate fetal dendritic and T cell interactions following in utero malaria exposure.

This study had a few notable limitations. First, malaria exposure prior to 12–20 weeks gestation was not assessed and thus the effect of very early in utero malaria exposure on fetal immune system development was not evaluated. In addition, the study was performed in a very high transmission region; more than half of the pregnant women were parasitaemic at the time of enrolment, and malaria infection was documented at some point in pregnancy or at delivery in nearly 80 % of subjects. Thus, the findings may not be generalizable to settings with lower transmission intensity and/or lower levels of pre-existing maternal immunity. Perhaps as a result of this high exposure intensity, most placentas showing histopathologic evidence of PM were categorized as ‘past’ rather than ‘active-acute’ infection [21]. This high ratio of past to active cases of PM may have been influenced by the use of intermittent preventive treatment in pregnancy (IPTp), as subjects in this study were enrolled in a chemoprevention trial; however, randomized treatment arm was not associated with any immunologic outcome measures. In study settings where more late-gestation PM infections occur, the impact on fetal T cell development may differ. Lastly, a number of immunologic and exposure outcomes were evaluated simultaneously and data were reported without adjustment for multiple comparisons introducing the potential for α-error inflation. Additional studies will be needed in order to validate these findings, and to determine whether the activated CD4 T cells described here represent malaria-specific responses.

## Conclusions

In summary, significant heterogeneity in T cell phenotypes were found to be related to the timing of in utero malaria exposure. T_regs_ were modestly increased in infants exposed to malaria early in gestation, whereas activated CD127^+^ CD4 T cells of an effector-memory phenotype were expanded in infants with active placental infection at the time of delivery. Fetal CD4 T cell differentiation is likely influenced by both the type and timing of antigen exposure. Prospective longitudinal studies are needed to determine the functional implications of fetal regulatory and effector CD4 T cells on the development of anti-malarial immunity during childhood.

## References

[1] Walker PGT, ter Kuile FO, Garske T, Menéndez C, Ghani AC (2014). Estimated risk of placental infection and low birthweight attributable to *Plasmodium falciparum* malaria in Africa in 2010: a modelling study. Lancet Glob Health.

[2] Desai M, ter Kuile FO, Nosten F, McGready R, Asamoa K, Brabin B (2007). Epidemiology and burden of malaria in pregnancy. Lancet Infect Dis.

[3] Schwarz NG, Adegnika AA, Breitling LP, Gabor J, Agnandji ST, Newman RD (2008). Placental malaria increases malaria risk in the first 30 months of life. Clin Infect Dis.

[4] Tonga C, Kimbi HK, Anchang-Kimbi JK, Nyabeyeu HN, Bissemou ZB, Lehman LG (2013). Malaria risk factors in women on intermittent preventive treatment at delivery and their effects on pregnancy outcome in Sanaga-Maritime, Cameroon. PLoS One.

[5] Le Port A, Watier L, Cottrell G, Ouédraogo S, Dechavanne C, Pierrat C (2011). Infections in infants during the first 12 months of life: role of placental malaria and environmental factors. PLoS One.

[6] Malhotra I, Dent A, Mungai P, Wamachi A, Ouma JH, Narum DL (2009). Can prenatal malaria exposure produce an immune tolerant phenotype?: a prospective birth cohort study in Kenya. PLoS Med.

[7] Flanagan KL, Halliday A, Burl S, Landgraf K, Jagne YJ, Noho-Konteh F (2009). The effect of placental malaria infection on cord blood and maternal immunoregulatory responses at birth. Eur J Immunol.

[8] Soulard V, Amadoudji Zin M, Fitting C, Ibitokou S, Oesterholt M, Luty AJF (2011). Placental malaria-associated suppression of parasite-specific immune response in neonates has no major impact on systemic CD4 T cell homeostasis. Infect Immun.

[9] Mackroth MS, Malhotra I, Mungai P, Koech D, Muchiri E, King CL (2011). Human cord blood CD4^+^ CD25hi regulatory T cells suppress prenatally acquired T cell responses to *Plasmodium falciparum* antigens. J Immunol.

[10] Brustoski K, Moller U, Kramer M, Hartgers FC, Kremsner PG, Krzych U (2006). Reduced cord blood immune effector-cell responsiveness mediated by CD4^+^ cells induced in utero as a consequence of placental *Plasmodium falciparum* infection. J Infect Dis.

[11] Nouatin O, Gbedande K, Ibitokou S, Vianou B, Houngbegnon P, Ezinmegnon S (2015). Infants’ peripheral blood lymphocyte composition reflects both maternal and post-natal infection with *Plasmodium falciparum*. PLoS One.

[12] Bisseye C, van der Sande M, Morgan WD, Holder AA, Pinder M, Ismaili J (2009). *Plasmodium falciparum* infection of the placenta impacts on the T helper type 1 (Th1)/Th2 balance of neonatal T cells through CD4(+)CD25(+) forkhead box P3(+) regulatory T cells and interleukin-10. Clin Exp Immunol.

[13] Ismaili J, van der Sande M, Holland MJ, Sambou I, Keita S, Allsopp C (2003). *Plasmodium falciparum* infection of the placenta affects newborn immune responses. Clin Exp Immunol.

[14] Mold JE, Michaelsson J, Burt TD, Muench MO, Beckerman KP, Busch MP (2008). Maternal alloantigens promote the development of tolerogenic fetal regulatory T cells in utero. Science.

[15] Debock I, Flamand V (2014). Unbalanced neonatal CD4(+) T-cell immunity. Front Immunol.

[16] Mold JE, McCune JM (2014). At the crossroads between tolerance and aggression. Chimerism.

[17] Rose S, Lichtenheld M, Foote MR, Adkins B (2007). Murine neonatal CD4^+^ cells are poised for rapid Th2 effector-like function. J Immunol.

[18] Kakuru A, Jagannathan P, Muhindo MK, Natureeba P, Awori P, Nakalembe M (2016). Dihydroartemisinin-piperaquine for the prevention of malaria in pregnancy. N Engl J Med.

[19] Hopkins H, González IJ, Polley SD, Angutoko P, Ategeka J, Asiimwe C (2013). Highly sensitive detection of malaria parasitemia in a malaria-endemic setting: performance of a new loop-mediated isothermal amplification kit in a remote clinic in Uganda. J Infect Dis.

[20] Natureeba P, Ades V, Luwedde F, Mwesigwa J, Plenty A, Okong P (2014). Lopinavir/ritonavir-based antiretroviral treatment (ART) versus efavirenz-based ART for the prevention of malaria among HIV-infected pregnant women. J Infect Dis.

[21] Rogerson SJ, Pollina E, Getachew A, Tadesse E, Lema VM, Molyneux ME (2003). Placental monocyte infiltrates in response to *Plasmodium falciparum* malaria infection and their association with adverse pregnancy outcomes. Am J Trop Med Hyg.

[22] Zhang X, Mozeleski B, Lemoine S, Deriaud E, Lim A, Zhivaki D (2014). CD4 T cells with effector memory phenotype and function develop in the sterile environment of the fetus. Sci Transl Med.

[23] Rissoan MC, Soumelis V, Kadowaki N, Grouard G, Briere F, de Waal Malefyt R (1999). Reciprocal control of T helper cell and dendritic cell differentiation. Science.

[24] Fang W-N, Shi M, Meng C-Y, Li D-D, Peng J-P (2016). The balance between conventional DCs and plasmacytoid DCs is pivotal for immunological tolerance during pregnancy in the mouse. Sci Rep.

[25] Michaelsson J, Mold JE, McCune JM, Nixon DF (2006). Regulation of T cell responses in the developing human fetus. J Immunol.

[26] Kassberger F, Birkenmaier A, Khattab A, Kremsner PG, Klinkert M-Q (2002). PCR typing of *Plasmodium falciparum* in matched peripheral, placental and umbilical cord blood. Parasitol Res.

[27] Xi G, Leke RGF, Thuita LW, Zhou A, Leke RJI, Mbu R (2003). Congenital exposure to *Plasmodium falciparum* antigens: prevalence and antigenic specificity of in utero-produced antimalarial immunoglobulin M antibodies. Infect Immun.

[28] Urban BC, Ferguson DJ, Pain A, Willcox N, Plebanski M, Austyn JM (1999). *Plasmodium falciparum*-infected erythrocytes modulate the maturation of dendritic cells. Nature.

[29] Breitling LP, Fendel R, Mordmueller B, Adegnika AA, Kremsner PG, Luty AJF (2006). Cord blood dendritic cell subsets in African newborns exposed to *Plasmodium falciparum* in utero. Infect Immun.

[30] Fievet N, Varani S, Ibitokou S, Briand V, Louis S, Perrin R (2009). *Plasmodium falciparum* exposure in utero, maternal age and parity influence the innate activation of foetal antigen presenting cells. Malar J.

[31] Encabo A, Solves P, Carbonell-Uberos F, Minana MD (2007). The functional immaturity of dendritic cells can be relevant to increased tolerance associated with cord blood transplantation. Transfusion.

[32] Ochando JC, Homma C, Yang Y, Hidalgo A, Garin A, Tacke F (2006). Alloantigen-presenting plasmacytoid dendritic cells mediate tolerance to vascularized grafts. Nat Immunol.

[33] Lombardi V, Speak AO, Kerzerho J, Szely N, Akbari O (2012). CD8alpha(+)beta(−) and CD8alpha(+)beta(+) plasmacytoid dendritic cells induce Foxp3(+) regulatory T cells and prevent the induction of airway hyper-reactivity. Mucosal Immunol.

[34] Ito T, Yang M, Wang Y-H, Lande R, Gregorio J, Perng OA (2007). Plasmacytoid dendritic cells prime IL-10-producing T regulatory cells by inducible costimulator ligand. J Exp Med.

